# Highlights from the Third International Central Nervous System Germ Cell Tumour symposium: laying the foundations for future consensus

**DOI:** 10.3332/ecancer.2013.333

**Published:** 2013-07-17

**Authors:** Matthew J Murray, Gail Horan, Stephen Lowis, James C Nicholson

**Affiliations:** 1 Department of Paediatric Haematology and Oncology, Addenbrooke’s Hospital, Cambridge CB2 0QQ, UK; 2 Department of Oncology, Addenbrooke’s Hospital, Cambridge CB2 0QQ, UK; 3 Department of Oncology, Bristol Royal Hospital for Children, Bristol BS2 8BJ, UK

**Keywords:** Third International CNS GCT Symposium, central nervous system, germ cell tumour, germinoma, nongerminoma

## Abstract

The Third International Central Nervous System (CNS) Germ Cell Tumour (GCT) Symposium brought together over 100 delegates from all over the world to learn about the latest developments in these tumours and discuss future strategies for their management. Some areas of consensus were agreed upon, and controversies were discussed. Among these, the classification of GCTs and the surgical approach to their management were among the greatest areas of difference between different parts of the world. The need for radiotherapy (RT) as a part of standard first-line management for all malignant CNS GCTs was agreed, as well as the need for additional chemotherapy to maximise the cure in nongerminomatous malignant GCTs; the benefit of the addition of chemotherapy in localised germinoma to reduce the RT burden was also accepted as a good practice. The potential of biological parameters to assist the future diagnosis, treatment stratification, and disease monitoring for CNS GCTs was discussed. Such biological parameters may also represent targets for the development of novel therapies. The need for further collaboration between groups engaged in biological studies was agreed. The merits of proton beam RT were debated, and the importance of mitigating the long-term side effects of the treatment was underlined by a session on late effects.

## Introduction

Germ cell tumours (GCTs) of the central nervous system (CNS) comprise a rare and histologically heterogeneous group of predominantly midline tumours for which optimal management involves the input of multiple disciplines, in view of the complexities of surgical access, availability of markers for diagnosis of some cases, response to both chemotherapy and radiotherapy (RT), and the frequent endocrine complications of both tumour and treatment. The evolution of treatment in the hands of neurosurgeons, radiation oncologists, and medical or paediatric oncologists has led to diverse approaches, which have been highlighted and discussed at previous meetings. The last ten years have seen some convergence of approaches, but a number of recognised challenges remain. These include the following:

(a) The timing and extent of surgery, both at prediagnosis and for residual disease identified during/following therapy.
(b) The most appropriate classification system for germinoma and nongerminoma (NGGCT).
(c) How little RT and chemotherapy are actually required to cure localised germinoma, without resulting in increased relapse rates.
(d) The optimal treatment approach for NGGCT and mixed malignant tumours.
(e) The optimal treatment approach for high-risk and relapsed disease.
(f) The need to increase our understanding of CNS GCT biology and exploit it prospectively in clinical trials.
(g) Identification of strategies to best mitigate against the long-term side effects of treatment.

There have now been three international symposia dedicated to the subject of the management of CNS GCTs, with the first in Kyoto, Japan, in 2003. Its aims were to understand more about the natural history of CNS GCTs, evaluate clinical treatment results, and facilitate the establishment of standard therapeutic protocols. The programme objectives for the second symposium, held in Los Angeles, United States, in 2005 were broadly educational, developing the themes of the first meeting. A number of the key controversies in the management of CNS GCTs were highlighted during the second meeting.

The aims of the third symposium were to further increase our understanding of CNS GCTs, by learning from both clinical and biological experiences from around the world, to overcome differences where necessary and to reach consensus where possible. Specifically, the academic sessions were structured to facilitate the discussion of the above questions, to explore the controversies, and conclude with round table discussions designed to lay the foundations for future consensus.

The symposium was organised by the UK’s Children’s Cancer and Leukaemia Group (CCLG) and was held at the University of Cambridge, in the United Kingdom, attracting a total of 117 delegates from 25 countries spread across five continents, representing all relevant multidisciplinary specialities. A significant strength of the symposium was that it succeeded in bringing together so many internationally recognised experts in one place. This report highlights some of the key areas discussed [1].

## Overview from three continents

Masao Matsutani from Saitama Medical Centre, Japan, introduced the first session with an overview of the treatment of CNS GCTs in Japan over the last 50 years, underlining the improvements in outcomes observed over time. Three risk groups are identified in Japanese treatment stratifications: (a) pure germinoma, (b) intermediate-prognosis GCTs, and (c) poor-prognosis GCTs, the latter two groups comprising mixed malignant tumours. Early studies were only single-institution reports but still demonstrated the efficacy of carboplatin and etoposide for the initial control of germinoma. In addition, they highlighted the need for extended local field RT, to avoid an excess of ventricular relapses. Now, RT fields including the ventricles are a widely accepted approach for germinoma. Results for poor-prognosis cases were generally unfavourable, although the treatments delivered were variable. The first prospective Japanese study, which recruited until 2003, showed ten-year progression-free survival (PFS) and overall survival (OS) of 80.4% and 96.4%, respectively, in 115 germinoma patients, with almost identical results for 35 additional cases singled out for high human chorionic gonadotropin (HCG) secretion. Results for intermediate- and poor-prognosis cases were inferior to those of germinoma, but improved on previous series—survival figures were compromised for poor-prognosis patients by reluctance to risk the toxicity of the concurrent chemotherapy and RT prescribed in the protocol. Important lessons from the study included confirmation of the need for extended field RT, inferior outcomes for basal ganglia germinoma (thus justifying whole brain RT for this group), salvageability of pure germinoma with chemo-RT following relapse, the importance of achieving complete remission (CR) in the intermediate group, and the need for both chemo and RT in high-risk patients.

Stewart Goldman (Chicago, United States) outlined the strategies followed in North America, including those with international collaborators. He discussed the lessons learned from key limited institution studies for germinoma, including the scope for response-based RT following chemotherapy, and the finding that chemotherapy-alone strategies were inferior to RT-containing regimens. The ACNS 0122 study (~100 patients) examined the benefit of second-look surgery for NGGCT patients not in CR after chemotherapy, followed by consolidation with high-dose chemotherapy. They also correlated tumour markers with radiological response and employed carboplatin rather than cisplatin-based chemotherapy regimens. Outcomes were good, with 84% and 93% event-free survival (EFS) and OS at five years, respectively. He highlighted the importance of incorporating biology into future trials and outlined the beginnings of collaborative approaches in the management of recurrent CNS GCTs.

Watch Stewart Goldman’s talk here http://ecancer.org/video/1963/overview-of-germ-cell-tumour-research-in-north-america.php

Finally, Gabriele Calaminus (Münster, Germany) presented the experience of the last 30 years from Europe. She summarised the questions posed by sequential German, French and ultimately, pan-European trials in relation to germinoma and nongerminoma. For germinoma, the sequential reduction of RT dose has been explored, along with the success of combination with chemotherapy and the indications and consequences of surgery. The RT dose was successfully reduced in the MAKEI (86/89) studies, such that EFS over 90% was achieved in the most recent trial (SIOP CNS GCT 96) for germinoma treated with craniospinal (CSI) RT. Combined treatment was found to be feasible using carboplatin, etoposide, and ifosfamide, but use of focal RT fields only left the ventricles at risk for relapse. Chemotherapy alone had also been tried with similar results to those of the international/US studies, with unacceptably high recurrence rates. For NGGCTs, the questions centred around the practicalities of diagnosis by markers alone, optimal chemotherapy agents and dose, and the roles of second-look surgery and spinal RT. The now standard European chemotherapy strategy with cisplatin, etoposide, and ifosfamide (‘PEI’) was established following a study identifying a total cisplatin dose of 400 mg/m^2 ^as superior to 200 mg/m^2^, followed by a successful pilot of PEI. The CNS GCT 96 study established the feasibility of diagnostics with radiology and tumour markers alone, resulting in avoidance of primary biopsy/ resection without compromise to survival. In addition, the importance of second-look surgery in patients who were less than CR after PEI chemotherapy, and the association of high alpha-fetoprotein (AFP) levels (>1000 ng/ml in either compartment) with poor prognosis were highlighted. EFS of localised disease treated with focal RT following chemotherapy was >70%, with predominantly local failures, supporting the strategy of sparing spinal fields in such patients, if fully staged at diagnosis.

## Epidemiology of CNS GCTs

Eric Bouffet (Toronto, Canada) provided an excellent overview of the epidemiology of this tumour group, offering several new insights. Data from the international (IARC) and North American (SEER-CBTRUS) databases showed overall incidence varying from <0.5/million in Israel, to 0.6/million in the United States, 1/million in the United Kingdom and Germany, to 2.7/million in Japan. There has been a threefold increase in incidence in children <15 years of age recorded in the SEER database in the 20 years from 1975–1980 to 1993–2000, and a twofold increase in young people aged 15–29 years. A controversial recent paper was discussed in which a similar incidence in Japan and North America was proposed based on analysis from two population-based and two hospital-based studies in each country. A literature review including a total of 3816 patients since 2000 broadly supported the accepted views of the geographical, age, and gender distribution and the distribution of site between pineal (37%–66%), suprasellar (23%–35%), and basal ganglia (0%–8%), with metastatic cases accounting for 5%–35%. The literature review suggested a more marked gender imbalance than suggested by the SEER/IARC analysis. A number of genetic risk factors have been proposed, many based on single case reports, but clear links have been demonstrated to Klinefelter’s and Down’s syndrome, in which GCTs are the most common type of brain tumour. Genetic, ethnic, and environmental factors, as well as population migration, were discussed as explanations of geographical variation in incidence. One particularly interesting report related organo-chlorine water pollutants to the development of germinoma in lake whitefish. In summary, the epidemiology and aetiology of CNS GCTs remains unclear, but it must be considered to be due to a complex interplay of multiple factors. The difficulties of drawing clear conclusions from data derived from multiple sources were underlined, and the need for harmonisation of data collection was proposed.

Finally, Keishi Makino (Kumamoto, Japan) outlined the results from Kumamoto (population 1.83 million) in Japan, confirming a higher incidence of CNS GCTs compared with China/Western countries.

Watch Eric Bouffet’s talk here http://ecancer.org/video/1966/epidemiology-of-central-nervous-system-germ-cell-tumours.php

## Diagnostics and presentation of CNS GCTs

The session was opened with a comprehensive overview of current imaging of CNS GCTs by Monika Warmuth-Metz (Würzburg, Germany), who has taken a leading role in both the German ‘HIT’ and European SIOP studies over the last 20 years. This was a masterclass in the use of computed tomography (CT) and magnetic resonance imaging (MRI) density in diagnosis, with particular reference to potential differential diagnoses such as low-grade glioma, craniopharyngioma, Langerhans cell histiocytosis, hypophysitis, and sarcoidosis. The strategy and process for real-time radiology review in the new SIOP CNS GCT II trial was outlined as an integral component of the aim to demonstrate the efficacy of reduced dose RT in responsive disease. Finally, the relative lack of information supporting the routine use of functional imaging techniques was highlighted as an area for future research.

Yuko Wanatabe (Saitama, Japan) described the use of methionine-PET in the diagnosis and monitoring of basal ganglia GCT, which are more difficult to diagnose by conventional imaging methods. John-Paul Kilday (Toronto) explored the relevance of the loss of the posterior pituitary ‘bright spot’ in the assessment and monitoring of suprasellar germinoma; it is absent in the majority of cases, particularly those presenting with diabetes insipidus. Since it relates to the functional integrity of the pituitary, its persistence in a minority is unexplained.

Juliet Hale (Newcastle, United Kingdom) presented data on 68 patients over ten years from four paediatric centres in three countries (Canada, the United Kingdom, and Germany), and Roshan Sethi (Boston, United States) on 70 patients treated in Boston from 1998 to 2012. Common themes identified included the tendency to long symptom intervals in GCTs (especially germinoma), greater delays in the diagnosis of suprasellar GCTs than pineal, and a preponderance of endocrine symptomatology, including diabetes insipidus, amongst those patients with the greatest delays. Delayed diagnosis was associated with metastatic disease at diagnosis but had no adverse impact on survival outcome, although the impact of delayed diagnosis on morbidity was highlighted in both series, with profound long-term consequences for patients. Factors contributing to delays came from both patients and physicians, with a need identified to target primary and secondary care.

Watch Juliet Hale’s talk here http://ecancer.org/video/1960/time-to-diagnosis-for-intracranial-germ-cell-tumours--possible-consequences-and-potential-for-improvement.php

Didier Frappaz (Lyon, France) presented experience of visual impairment in germinoma in a cohort of patients from Lyon. In addition to the well-recognised upward-gaze paresis associated with pineal tumours, this series of 28 patients highlighted the effects on visual acuity from papilloedema and from optic nerve involvement in a small number of cases. He stressed the need to look closely for compression and invasion of optic nerves in any patients with germinoma and visual impairment, with appropriate extension of RT fields.

The session closed with a keynote presentation by Ivo Leuschner (Kiel, Germany) outlining the histological classification of CNS GCTs. The immunohistochemical markers in current use include CD117/KIT, POU5F1 (OCT3/4), PLAP, and CT45 (germinoma); AFP, SALL4 and glypican 3 [yolk sac tumour (YST)]; CD30 (embryonal carcinoma); and HCG (choriocarcinoma). However, it was cautioned that there may be some overlap between the expressions of these markers. Consequently, pathological review by a histopathologist experienced in these tumours was encouraged, with central review becoming more common in current clinical trials.

Watch Ivo Leuschner’s talk here http://ecancer.org/video/1958/classification-and-immunohistochemical-approach-of-intracranial-germ-cell-tumours.php

## Biology of GCTs

Previous sessions had highlighted the potential importance of tumour biology in helping to understand the pathogenesis of CNS GCTs and for future incorporation into clinical trials. However, due to the rarity of the disease and the paucity of tumour specimens available, very few basic research studies have been performed to date. The Northern Hemisphere approach, where positive tumour markers obviate the need for neurosurgical biopsy, has exacerbated this lack of available material for studies. Where neurosurgical biopsy is undertaken, the specimen sizes left over for research after diagnostic studies are completed are often very small, limiting the range of biological studies that can be performed. However, delegates at the symposium heard how significant progress had been made in this area.

Koichi Ichimura (Tokyo, Japan) and Ching Lau (Houston, United States) opened the session with complementary presentations of their studies of whole exome sequencing (WES) in CNS GCTs. Both speakers described their established consortia which had collected a substantial number of biological specimens and associated clinical data. A proportion of these specimens had undergone WES, which selectively sequences the protein-coding regions (exons) of the genome and can therefore identify mutations in key protein-coding genes, which may underlie tumour development. Somatic KIT and RAS mutations were described, which were mutually exclusive, and which may assist the development of targeted therapy for resistant tumours. Novel germline mutations that are likely to provide insights into the genetic predisposition of this disease were also identified. Keita Terashima (Tokyo), working with Ching Lau’s group, then presented data on DNA copy-number changes in CNS GCTs. Specimens were analysed for copy-number alterations and loss of heterozygosity, using single nucleotide polymorphism array and qRT-PCR. Informative regions of gain and loss were identified, with overall patterns similar between germinoma and NGGCT. In addition, they found some histological subtype specific changes.

Matthew Murray (Cambridge) described a global microRNA profiling study in GCTs. MicroRNAs are short, nonprotein-coding RNAs which negatively regulate protein-coding gene expression. All malignant GCTs overexpressed the miR-371~373 and miR-302 clusters, regardless of patient age (paediatric/adult), tumour site (intracranial/extracranial), or histological subtype (germinoma/YST/embryonal carcinoma). He demonstrated the translational potential of this finding, since microRNAs from these two clusters were present at high levels in patient serum at the time of extracranial malignant GCT diagnosis, with levels of miR-372 falling following treatment. Importantly, miR-371~373 and miR-302 cluster serum microRNA levels were high at diagnosis in patients with (AFP/HCG) marker-negative disease.

Watch Matthew Murray’s talk here http://ecancer.org/video/1961/global-microrna-profiles-in-extracranial-and-intracranial-malignant-germ-cell-tumours.php

Keita Terashima extended these observations, showing that levels of these microRNAs were elevated in the cerebrospinal fluid (CSF) when malignant CNS GCT diagnosis, including in AFP/HCG marker-negative germinomas. CSF levels of these microRNAs at diagnosis were significantly higher than those following treatment. In the future, it will be important to see if CSF microRNAs may reliably distinguish germinoma from NGGCTs, as treatment strategies are different. The prospective study of CSF levels of miR-371~373 and miR-302 clusters and other subtype-specific microRNAs is now warranted in large patient cohorts.

## Free papers—clinical reports and trials

Submitted papers on first-line treatment of germinoma generally described combined treatment with chemotherapy followed by RT, the focus being on sparing long-term toxicity, a recognised risk of proven RT-only approaches.

Results of the Los Angeles Children’s Hospital germinoma series from 2003 to 2008 were presented by Jonathan Finlay. Although this was a single-institution study with only 21 patients, the uniform approach to management and inclusion of comprehensive toxicity data, particularly on neurocognitive outcomes, to complement survival figures, made this a particularly valuable report. Four courses of carboplatin and etoposide were given, followed by ventricular field RT of 21.4–24 Gy, with a boost to the tumour (total dose 30 Gy). Responses to chemotherapy were generally excellent, with PFS and OS at seven years of 91.5% and 96.5%, respectively. There were no adverse impacts on IQ, memory, or executive function at 4.4 years from diagnosis. The relapse of one case as a mixed malignant GCT with raised AFP, having had a minimally raised AFP of 2.9 ng/ml at first diagnosis, raised the question of whether any AFP rise should be acted upon, regardless of biopsy findings and in contrast to most cutoffs used to define NGGCT.

Watch Jonathan Finlay’s talk here http://ecancer.org/video/1964/treatment-of-primary-central-nervous-system-germinoma-with-chemotherapy.php

Geneviève Legault (New York, United States) described the outcomes following chemotherapy and involved field RT in 23 localised germinomas from two combined US series. Although the relapse rate (4/23) was higher than reported in RT-only series, all were salvaged, and the relapse pattern suggested that extension of fields to whole ventricles would not have prevented recurrence.

Adam Schoenfeld (San Francisco, United States) presented the University of California, San Francisco germinoma experience over 30 years from 1980 to 2010. A total of 79 patients were treated with either RT alone or combinations of chemotherapy with focal or whole ventricular (WVRT). Survival was significantly inferior for those receiving focal RT only and suggested a higher risk of recurrence for cases with HCG > 50 IU/l.

Claire Alapetite (Paris, France) compared presentations and outcomes in young adults with those of adolescents treated on the French TGM-90 protocol. She reported the tendency to more advanced disease at presentation in patients >18 years at diagnosis, and poor compliance with follow-up. She discussed her findings in the context of the particular needs of adolescents and young adult patients, the importance of psychosocial assessment and support, and highlighted the need for dedicated multidisciplinary teams to care for these patients in appropriate settings.

Three papers focused on the issues presented by bifocal tumours. Lucie Lafay-Cousin (Calgary, Canada) presented a review of 16 patients from two Canadian centres, Calgary and Toronto. Fifty-six per cent were diagnosed without biopsy, 94% received chemotherapy, and RT fields were WVRT in 50% and focal or CSI in the rest. Two relapses were seen, both in the focal RT group. The authors concluded that chemotherapy followed by WVRT is sufficient for treatment of these tumours, supporting the classification of bifocal tumours as loco-regional rather than metastatic disease. Shannon Macdonald (Boston) presented Boston data in which a small number of bifocal NGGCT were identified, with three biopsy proven cases including YST elements in patients who had mildly raised AFP levels, but which were below ‘secreting’ thresholds. Therefore, she advised caution over diagnosis of bifocal germinoma without biopsy in such cases, and repeating markers if borderline.

Hee Jo Baek (Gwangju, Korea) presented the results of a national Korean study of 92 patients with NGGCT diagnosed from 2005 to 2011 who received intensive chemotherapy including carboplatin, cyclophosphamide, etoposide, and bleomycin, followed by CSI RT, with encouraging early results. With a median follow-up of 41 months, the five-year EFS and OS were 81.8% and 93.6%, respectively. Veronica Biassoni (Milan, Italy) presented the Milan experience of 24 NGGCT patients receiving BEP (bleomycin, etoposide, cisplatin) chemotherapy followed by CSI RT, with 78% and 79% PFS and OS, respectively, similar to that of the large international studies such as SIOP CNS GCT 96. They identified high-risk patients for relapse with high AFP and proposed intensification with high-dose chemotherapy for this group. Girish Dhall (Los Angeles) presented four cases of CNS GCT in Down’s syndrome and noted their ability to tolerate standard treatment and also high-dose chemotherapy, with good outcomes.

James Nicholson (Cambridge) presented a subgroup of 59 patients (from a total of 190) treated as NGGCT in the recent SIOP CNS GCT 96 study, who had raised serum or CSF HCG as the basis for diagnosis. Outcomes for 39 patients with HCG 50–200 IU/l were superior to those for 20 cases with HCG > 200 IU/l: no recurrences or events were seen, compared with PFS and OS of 72% and 87%, respectively, if HCG was >200 IU/l. Where histology was available, the majority of malignant components were germinoma, and only four cases had evidence of choriocarcinoma. The presentation stimulated debate on the most appropriate cutoff for HCG to classify as NGGCT for treatment and a general recognition that 50 IU/l was likely to result in at least some germinomas being overtreated.

The proposed new North American (COG) studies (ACNS 1123) for both localised germinoma and nongerminoma were discussed by Ute Bartels (Toronto) and Jason Fangusaro (Chicago). For germinoma, the aim is to evaluate a simplified (carboplatin and etoposide based) chemotherapy regimen followed by response-based RT. For NGGCTs, the focus is also on reduction of RT for chemo-responsive cases, using a carboplatin-based strategy. Both strata have inbuilt prospective evaluations of cognitive, social and behavioural function.

## Surgical management of CNS GCTs

Thomas Czech (Vienna, Austria) opened the session by outlining the European surgical management of CNS GCTs. Germinoma cases require neurosurgical biopsy to obtain a histological diagnosis. Following the treatment, there was no difference in five years PFS or EFS when comparing patients with a residual lesion and those without it. For NGGCTs, 50% of cases still underwent histological confirmation, despite typical radiology and raised CSF and/or serum AFP/HCG markers being sufficient for diagnosis. Patients with pineal NGGCTs who had a nonresected residual mass following chemotherapy, but before RT, had significantly more events than those patients in whom the residual was resected, and hence second-look surgery in such cases is warranted. The impact of this strategy is less clear in patients with an NGGCT in the suprasellar region and here the relative merits/risks of surgery need to be carefully considered on an individual basis.

Christian Dorfer (Vienna) gave an overview of the different surgical corridors to approach pineal tumours, including GCTs, and how the risks and benefits of each approach need to be individually adapted for the patient. Benedetta Pettorini (Liverpool, United Kingdom) and Mitsuteru Oikawa (Sapporo, Japan) provided an update on neuroendoscopic approaches in CNS GCT management. This included endoscopic third ventriculostomy as the favoured management for hydrocephalus caused by pineal region tumours, where feasible. Neuroendoscopic biopsies were also possible in the majority of cases, where indicated.

Tai-Tong Wong (Taipei, Taiwan) gave a comprehensive overview of the management of intracranial teratoma, comprising 4% of all CNS GCTs, which relies predominantly on complete surgical resection. He commented that the natural history of immature teratoma (IT) in patients <1 year of age is substantially inferior to those >1 year, and consequently, where residual tumour remains following initial surgery, second-look surgery with the aim of complete resection should be attempted to salvage such patients. Both Jeffrey Murray (Fort Worth, Texas, United States) and Tamio Ito (Sapporo) highlighted the problem of growing teratoma syndrome in IT cases.

Conor Mallucci (Liverpool) gave an informative overview detailing recent surgical advances and challenges for these tumours. He highlighted the ability to undertake neuroendoscopic biopsies in the majority of cases. In addition, the use of intraoperative MRI offers a number of key advantages, although it adds an additional 1–1.5 h to each procedure. It assists the neurosurgeon in ensuring a complete resection, removes the need for a further postoperative MRI, which often requires further sedation or general anaesthetic, and in cases where a residual is identified, avoids further repeat surgery.

Rames Kirollos (Cambridge) highlighted the frequent occurrence of complex disorders of accommodation and convergence following the treatment of tumours of the pineal gland, which included GCTs, for which corrective prisms were only partially effective. Finally, Mark Souwedaine (New York) presented potential neurosurgical complications. He highlighted that the risk profile for suprasellar and pineal region tumours were not generally dependent on tumour type. Biopsy-related haemorrhage, iatrogenic tumour dissemination (along surgical corridors or via shunts) and erroneous tissue sampling were discussed. The risk profile associated with second-look surgery generally supports continued implementation of this strategy for residual disease, particularly for pineal NGGCT cases.

## RT approaches in the management of CNS GCTs

CSI RT has traditionally been regarded as the gold standard treatment for germinoma, but has long-term sequelae. Most recent strategies have involved the use of chemotherapy to reduce the volume irradiated and the dose delivered, demonstrating that this approach can safely reduce the burden of radiation without compromising OS. For NGGCT, a radiation dose over 50 Gy is needed following chemotherapy to maximise the chance of a cure, and European trials have shown that CSI irradiation can be avoided and replaced with involved field radiation for fully staged localised NGGCT.

The central focus of the RT session was the role of new modalities of treatment. Daniel Holyoake (Cambridge) described the technique and benefits of intensity-modulated RT using tomotherapy, whilst Roshan Sethi reviewed 13 years of experience with proton therapy in Boston involving 70 patients with pure germinoma and NGGCT.

The highlight of the session was a debate of the motion ‘This house believes that all children and young people with intracranial GCTs should be offered proton therapy,’ which proved to be both entertaining and informative. Claire Alapetite and Shannon Macdonald (Boston) presented the rationale for proton beam RT in the context of European and North American GCT series. The European data illustrated the potential benefit from utilising a mode of therapy which minimised dose to normal tissue, whilst the efficacy of involved field RT for localised NGGCT could be used to justify attempts to further limit the dose to surrounding tissues through protons. They presented early data from Boston, suggesting that the outcomes with protons compared favourably with conventional photon-based RT. Other possible benefits cited included allowing reduction of a) chemotherapy in germinoma and b) late effects of treatment, including second malignancies. It was suggested that the additional cost of protons for GCT treatment would be mitigated by later health economic savings from lower lifetime morbidity. The value of protons for the management of GCTs was opposed by Thomas Merchant (Memphis, United States) and Michael Williams (Cambridge). Central to their arguments were the excellent quality of survival and neurocognitive outcomes now demonstrated with the most advanced photon techniques and the similarity of proton and photon techniques in terms of the high-dose volume treated. They also suggested that the technology for localisation and verification of protons lagged behind that for photons, with consequent uncertainties about exact proton dosimetry. Concerns regarding the impact of total body neutron dose on second malignancies were also raised. Finally, they stated that more data to demonstrate superior outcomes with protons need to be published before it can become routinely adopted for all children and young people with CNS GCTs. The motion was defeated both before and after the debate by 15 votes.

## Management of high-risk and relapsed disease

Significant progress has been made since the second symposium regarding the more organised management of high-risk and relapsed CNS GCTs. Whilst there are still no multicentre trials of second-line treatment yet to report, patterns of high-risk disease are more clearly identified and common themes to the approach to relapsed disease are emerging.

Gabriele Calaminus presented data on the high-risk groups within the NGGCT group of the SIOP CNS GCT 96 trial. Two risk factors have been identified as significant predictors for recurrence: the presence of residual disease at the end of treatment, and AFP > 1000 ng/ml at diagnosis. The high-AFP group did not have an excess of residuals to explain their poor outcomes (PFS 40%), and treatment for this group in SIOP CNS GCT II is being intensified.

The question of late recurrences of GCTs, within and beyond the CNS, and whether these are in fact metachronous GCT occurrences was discussed in a presentation delivered by Nicole Flores (Los Angeles), where cases recurring up to 17 years from first diagnosis were described, underlining the need to consider the possibility of underlying genetic susceptibility and provide long-term follow-up for these patients.

A number of series of relapsed patients were presented and potential relapse strategies discussed, on the premise that optimum treatment in this situation is unknown. Direct comparison of the results of the strategies presented is hampered by the heterogeneity of the patient groups reported, particularly in terms of the primary treatment given.

Matthew Murray presented relapses from the United Kingdom and Germany in patients who had received first-line treatment according to the SIOP CNS GCT 96 protocol. He reported 13 relapsed germinomas, including those treated initially with CSI RT and those who had received chemotherapy and focal RT. Relapse management included high-dose chemotherapy and autograft (HD + ASCT) for some patients. Approximately half were salvaged: there was no definite survival advantage of HD + ASCT in first relapse but data support a strategy of standard chemo and RT at first relapse with HD + ASCT at second.

Forty-five patients with relapsed NGGCT were reviewed, all after receiving PEI chemotherapy and RT according to dissemination. Salvage rates were poor, with no patients salvaged without HD + ASCT and only three long-term survivors out of 18 who received HD + ASCT.

Jonathan Finlay (Los Angeles) presented 14 cases of relapsed germinoma, 13 of whom received HD + ASCT at relapse. Outcomes were excellent, with over 90% salvage, and it was suggested that marrow-ablative chemotherapy at recurrence may afford lessened neurotoxicity through reduction or elimination of reirradiation. Of 21 cases of mixed malignant CNS GCT, over 80% were responsive to reinduction chemotherapy, with 54% of cases achieving minimal tumour status salvaged with HD + ASCT, without additional RT.

Kyung Duk Park (Seoul, Korea) reviewed outcomes of relapse in Seoul over 23 years. There were three survivors out of 13 relapsed cases, two germinoma, of which one was salvaged with HD + ASCT and one ‘teratocarcinoma,’ treated with gross total resection followed by chemotherapy. The potential of durable responses of relapsed GCTs to oral etoposide were demonstrated by Takaaki Yanagisawa (Saitama-Ken, Japan), who described a series of eight patients in which the treatment was well tolerated. The Los Angeles phase II trial of gemcitabine, paclitaxel, and oxaliplatin was described by Yin Liu. The combination shows promise as a reinduction regimen before high-dose therapy, even in heavily pretreated patients. The tolerability of the oral tyrosine kinase inhibitor, dasatinib, as an adjunct to conventional chemotherapy was explored by Diana Osorio (New York) in a North American study, but no data supporting efficacy were presented.

## Late effects of treatment, quality of survival, and neuropsychology

The final session was dedicated to early and late sequelae of CNS GCTs and their treatment. Gabriele Calaminus opened the session with a review of toxicity experienced by NGGCT patients treated in the European SIOP CNS GCT 96 trial. At presentation, symptoms of endocrine dysfunction, most commonly diabetes insipidus, were identified in almost half of the cases; neurological symptoms were seen in the majority, raised intracranial pressure predominating, and visual symptoms were seen in about two-thirds of cases.

Long-term toxicity consisted mainly of neuropsychological or neurocognitive impairments. Neurological impairments tended to improve with treatment, but long-term endocrinological disturbances were mainly related to impairments present at diagnosis.

Irene Slavc (Vienna) and her coworkers undertook a longitudinal study of neurocognitive outcomes in 24 consecutive patients treated in Vienna on the SIOP protocols and found that whilst IQ was preserved within the normal range, specific deficits were noted. These included working memory, visual spatial perception, and visual memory, which were all below average in about a third of cases. There was no apparent influence of site or tumour subtype. Stephen Sands (New York) gave an overview of late effects, outlining the current understanding regarding risk factors, particularly relating to RT. Reports of psychosocial and neurocognitive impairments have varied but generally suggested mild deficits at worst after treatment for GCTs, with physical deficits more marked than changes in IQ. Differences became more apparent when more specific deficits were investigated, with working memory and processing speed affected more with tumours at some sites than others. The inclusion of mandatory neurocognitive testing in the latest COG clinical trials is clearly a welcome step toward a greater understanding of this area.

Shih-Yuan Liang (Taipei) delivered an outstanding presentation describing neurocognitive outcomes and quality of survival in 56 cases of CNS GCT treated in Taiwan, focusing particularly on the impact of the tumour site and radiation field. She found consistently worse neuropsychological outcomes across most tests for patients with tumours of the basal ganglia compared with suprasellar and pineal regions, and similarly for whole brain or CSI compared with WVRT. Similar trends were seen for both site and RT field in quality of life measures. Memory disturbances were seen particularly in the pineal and basal ganglia cases, partly attributed to hydrocephalus and surgical approaches. Assunta Albanese (London, United Kingdom) ended the session with a comprehensive and informative overview of pituitary hormone replacement following treatment for CNS GCTs. She discussed the complexities of management of diabetes insipidus and adypsia, interactions between the cortisol axis and both thyroid and growth hormone axes, timing of growth hormone treatment, and the need for lifelong monitoring and treatment with appropriate transition arrangements in place.

## Summary and conclusion

Almost ten years have elapsed since the first symposium, and this has been reflected by substantial progress in the following areas:

(a) The understanding of the biology of CNS GCTs.
(b) Improvements in neurosurgical techniques.
(c) The development of more collaborative clinical trials to underpin future treatment strategies.
(d) The development of proton beam RT.
(e) More successful treatment of relapsed cases of CNS GCTs.

For germinoma treatment, the approaches followed by different groups are undoubtedly converging and, whilst management of NGGCT is more problematic and heterogeneous, reported outcomes have improved since the symposium in 2003. For relapsed malignant CNS GCTs, there are currently no open international trials recruiting patients. Despite this, more evidence was presented on this topic at the third symposium than ever before, and some common themes are emerging. Progress in biology has been marked with a move from isolated small studies of uncertain clinical significance to a coordinated multi-institutional approach with real potential for progress in clinical management. Quality of survival has been a focus of treatment strategies around the world, with greater potential for preservation of function from the most recent and planned treatments. A number of unanswered questions remain, and different historical backgrounds for the management of these cases on different continents make early full consensus on management unrealistic. Moreover, collaborations on a wider scale are hampered by differences in classification and lack of consistent approaches between groups in terms of diagnostics and surgical management. However, it was recognised that it should now be possible to agree on minimum standards which could help to achieve more effective and equitable treatment for children and young people with CNS GCTs around the world, in low income as well as developed countries, and that this should be a focus for future meetings.

## Acknowledgment

The authors would like to thank all those on the Scientific Advisory Board of the Third International CNS GCT Symposium for their input into the programme and all the presenters for their valuable contributions.

## Conflicts of interest

None to declare.

## Author contributions

All authors were involved in organising the symposium and were all members of the International Scientific Advisory Board. They all contributed in writing this article.
